# Development of A Radiomic Model for *MGMT* Promoter Methylation Detection in Glioblastoma Using Conventional MRI

**DOI:** 10.3390/ijms25010138

**Published:** 2023-12-21

**Authors:** Fabio M. Doniselli, Riccardo Pascuzzo, Massimiliano Agrò, Domenico Aquino, Elena Anghileri, Mariangela Farinotti, Bianca Pollo, Rosina Paterra, Valeria Cuccarini, Marco Moscatelli, Francesco DiMeco, Luca Maria Sconfienza

**Affiliations:** 1 Neuroradiology Unit, Fondazione IRCCS Istituto Neurologico Carlo Besta, 20133 Milan, Italy; fabio.doniselli@istituto-besta.it (F.M.D.); domenico.aquino@istituto-besta.it (D.A.); valeria.cuccarini@istituto-besta.it (V.C.);; 2 Department of Biomedical Sciences for Health, Università Degli Studi di Milano, 20133 Milan, Italy; 3 Post-Graduate School in Radiodiagnostics, Università Degli Studi di Milano, 20122 Milan, Italy; 4 Neuro-Oncology Unit, Fondazione IRCCS Istituto Neurologico Carlo Besta, 20133 Milan, Italy; elena.anghileri@istituto-besta.it (E.A.);; 5 Neuroepidemiology Unit, Fondazione IRCCS Istituto Neurologico Carlo Besta, 20133 Milan, Italy; mariangela.farinotti@istituto-besta.it; 6 Neuropathology Unit, Fondazione IRCCS Istituto Neurologico Carlo Besta, 20133 Milan, Italy; 7 Department of Neurosurgery, Fondazione IRCCS Istituto Neurologico Carlo Besta, 20133 Milan, Italy; francesco.dimeco@istituto-besta.it; 8 Department of Oncology and Hematology-Oncology, Università Degli Studi di Milano, 20122 Milan, Italy; 9 Department of Neurological Surgery, Johns Hopkins Medical School, Baltimore, MD 21205, USA; 10 Radiology Unit, IRCCS Istituto Ortopedico Galeazzi, 20157 Milan, Italy

**Keywords:** radiomics, *MGMT* promoter methylation, glioblastoma, neuro-oncology

## Abstract

The methylation of the O6-methylguanine-DNA methyltransferase (*MGMT*) promoter is a molecular marker associated with a better response to chemotherapy in patients with glioblastoma (GB). Standard pre-operative magnetic resonance imaging (MRI) analysis is not adequate to detect *MGMT* promoter methylation. This study aims to evaluate whether the radiomic features extracted from multiple tumor subregions using multiparametric MRI can predict *MGMT* promoter methylation status in GB patients. This retrospective single-institution study included a cohort of 277 GB patients whose 3D post-contrast T1-weighted images and 3D fluid-attenuated inversion recovery (FLAIR) images were acquired using two MRI scanners. Three separate regions of interest (ROIs) showing tumor enhancement, necrosis, and FLAIR hyperintensities were manually segmented for each patient. Two machine learning algorithms (support vector machine (SVM) and random forest) were built for *MGMT* promoter methylation prediction from a training cohort (196 patients) and tested on a separate validation cohort (81 patients), based on a set of automatically selected radiomic features, with and without demographic variables (i.e., patients’ age and sex). In the training set, SVM based on the selected radiomic features of the three separate ROIs achieved the best performances, with an average of 83.0% (standard deviation: 5.7%) for accuracy and 0.894 (0.056) for the area under the curve (AUC) computed through cross-validation. In the test set, all classification performances dropped: the best was obtained by SVM based on the selected features extracted from the whole tumor lesion constructed by merging the three ROIs, with 64.2% (95% confidence interval: 52.8–74.6%) accuracy and 0.572 (0.439–0.705) for AUC. The performances did not change when the patients’ age and sex were included with the radiomic features into the models. Our study confirms the presence of a subtle association between imaging characteristics and *MGMT* promoter methylation status. However, further verification of the strength of this association is needed, as the low diagnostic performance obtained in this validation cohort is not sufficiently robust to allow clinically meaningful predictions.

## 1. Introduction

Glioblastoma (GB) is the most common primary malignant brain tumor and one of the most aggressive and lethal neoplasms in adults, with a median survival of less than 15 months and a 5-year survival rate of 5.1% [1]. Among all identified genetic alterations, O6-methylguanine-DNA methyltransferase (*MGMT*) promoter (MGMTp) methylation remains the most important prognostic molecular marker in clinical settings [2]. *MGMT* encodes a DNA repair protein that directly detoxifies cytotoxic DNA damage resulting from the administration of chemotherapy [3]. The methylation of MGMTp is related to a better prognosis because of a greater effect of temozolomide (TMZ), the standard-of-care chemotherapy for GBs [4], when associated with radiotherapy [5,6]. The methylation of the MGMTp is associated with the suppression of gene expression. As such, MGMTp methylation confers an increased likelihood of a therapeutic response to TMZ [7]. Therefore, its detection could help in selecting patients who may benefit the most from TMZ therapy. However, discrepancies between MGMTp methylation status and treatment response can occur in some patients, probably due to the inconsistencies between MGMTp methylation and expression levels in GBs [8].

Magnetic resonance imaging (MRI) is widely accepted as the modality of choice for GB diagnosis and the evaluation of treatment response. However, the conventional evaluation of MRI predicts MGMTp methylation status poorly [9]. MGMTp-methylated GBs may show characteristics that are somewhat different from non-methylated GBs, as they seem to have mixed nodular enhancement, a limited amount of edema, higher apparent diffusion coefficient (ADC) minimum values, lower relative cerebral blood volume (rCBV) [10,11], and increased 18F-fluoro-2-deoxy-d-glucose (FDG) uptake with a comparable 11C-methionine (MET) uptake [12]. The results of these works based on the conventional MRI have not always been uniform, in some cases reaching values of statistical significance [13,14] and in others not [15,16].

Radiomics is an emerging discipline that aims to make predictions (such as diagnosis and prognosis) and derive medical treatment insights based on quantitative features extracted from medical images [17,18,19]. Radiomics is a multistep process that can be applied to any set of medical images, including both the conventional and more advanced MRI (e.g., diffusion tensor imaging, susceptibility-weighted imaging, and perfusion-weighted imaging) [20,21,22]. Radiomics can capture information about regions beyond the tumor borders and into the peritumoral space, where surgery does not normally extend [23,24].

Some studies have attempted to correlate MGMTp methylation status in GBs with radiomic features, but the results are contradictory [25,26]. These studies often showed profound differences in methodology and in patients’ characteristics, making the comparison of these results difficult.

In the present retrospective study, we aim to develop a predictive model of MGMTp methylation status in GBs based on radiomic features extracted from the entire tumor on pre-operative multiparametric magnetic resonance images (i.e., 3D T1-weigthed gadolinium contrast-enhanced (T1c) and 3D fluid-attenuated inversion recovery (FLAIR) images). We used two of the most widely employed machine learning algorithms to develop predictive models based on radiomic features: support vector machine (SVM) and random forest (RF) [27,28]. SVM is a powerful algorithm for supervised learning that works by finding the hyperplane that optimally separates data into distinct classes, while maximizing the margin between them. SVM is proficient in high-dimensional spaces, and kernel functions enable it to manage non-linear relations. RF is an ensemble learning approach that creates several decision trees during training and then provides the mode of the classes of the individual trees as the output. RF reduces overfitting and enhances accuracy by combining predictions from diverse decision trees. In addition, we investigate whether models based on the radiomic features extracted from singular tumoral subregions (necrosis (NEC), contrast enhancement (CE), or T2 hyperintensity (HYP)) or their combination provide a higher diagnostic accuracy than those based on the entire tumor analyses.

## 2. Results

**Figure 1:**
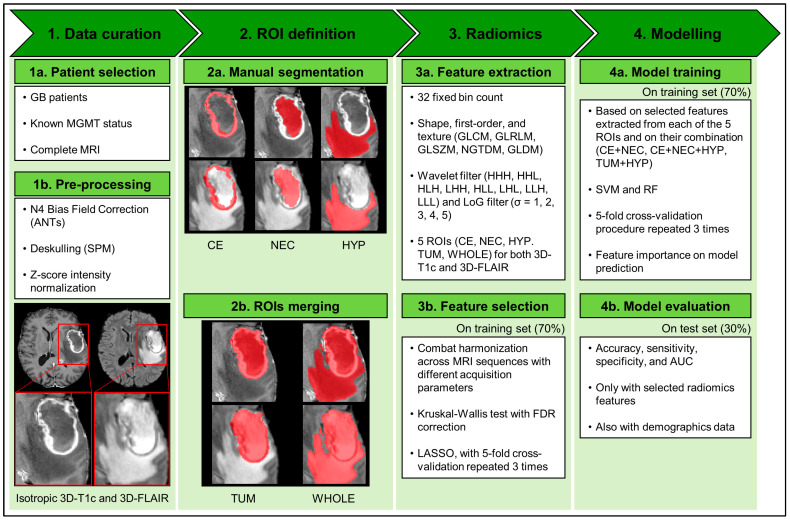
Analysis pipeline. Steps 1a–3a were conducted on the whole dataset, steps 3b and 4a only on the training dataset (70% of the whole dataset), and step 4b only on test dataset (the remaining 30% of the whole dataset). Abbreviations: GB = glioblastoma; MGMT = O6-methylguanine-DNA methyltransferase; MRI = magnetic resonance imaging; ANTs = advanced normalization tools; SPM = Statistical Parametric Mapping; 3D-T1c = 3D T1-weighted imaging gadolinium contrast-enhanced; 3D-FLAIR = 3D fluid-attenuated inversion recovery; ROI = region of interest; CE = contrast enhancement; NEC = necrosis; HYP = peritumoral T2-hyperintensity on fluid-attenuated inversion recovery image; TUM = tumor core (union of CE and NEC); WHOLE = whole tumor (union of CE, NEC, and HYP); GLCM = gray-level co-occurrence matrix; GLRLM = gray-level run length matrix; GLSZM = gray-level size zone matrix; NGTDM = neighboring gray tone difference matrix; GLDM = gray-level dependence matrix; H = high-pass filter of wavelet transformation in one of three spatial directions; L = low-pass filter of wavelet transformation in one of three spatial directions; LoG = Laplacian of Gaussian; FDR = false discovery rate; LASSO = least absolute shrinkage and selection operator; SVM = support vector machine; RF = random forest; AUC = area under the curve.

The pipeline used for the data extraction and analysis is shown in Figure 1 and consisted of the following steps: (i) the images were collected and pre-processed; (ii) the regions of interest (ROIs) identifying tumor subregions were manually segmented; (iii) the radiomic features were extracted from each sequence and subregion, harmonized and selected; and (iv) two classification algorithms were trained and validated based on the harmonized selected features. A detailed description of each step is provided in Section 4.

We included 277 MRI studies comprising 3D-T1c and 3D-FLAIR images out of the overall 292 selected patients with IDH-wildtype GBs. The exclusion of the remaining 15 was based on the lack of the necrotic component (10/292, 3.4%) or the absence of the peripheral hyperintense changes in FLAIR (5/292, 1.7%). Among the included patients, 121 (43.7%) had MGMTp-methylated and 156 had (56.3%) MGMTp-unmethylated tumors. The patients were subsequently assigned to a training set (*n* = 196) and a test set (*n* = 81). The baseline characteristics of the included patients are summarized and compared between the training and test sets in Table 1. There were no significant differences in patient and tumor characteristics between the two sets (*p*-value ranged from 0.225 to 0.999). There were no missing data in the dataset, except for KPS, which was not available in 19 and 8 patients of the training and test sets, respectively. KPS was not used for developing the models.

### 2.1. Selected Radiomic Features

In total, 2562 radiomic features were extracted for both MRI modalities (3D-T1c and 3D-FLAIR) with and without filtering, and encompassed shape, first-order, and texture features. These features were obtained for each of the three ROIs (CE, NEC, and HYP) representing singular tumoral subregions, for the tumor core (TUM, obtained by merging the CE and NEC components), and for the whole tumor (WHOLE, obtained by merging the CE, NEC, and HYP components).

After feature harmonization performed using the ComBat technique, we excluded between 34 and 77 radiomic features based on the ROI considered for the analysis because they showed statistically significant differences among groups of sequences in the training set (Supplementary Table S1) according to Kruskal–Wallis test. Of note, there were four texture features extracted from both T1 and FLAIR that were excluded from the set of candidate features for every ROI: GLSZM large area high gray-level emphasis, GLSZM large area low gray-level emphasis, GLSZM zone variance, and GLSZM large area emphasis.

The remaining sets of features were further selected through the least absolute shrinkage and selection operator (LASSO) technique. In total, based on the ROI considered for the feature extraction, between 41 and 151 radiomic features were used as input for the two machine learning algorithms (CE: 43 features; NEC: 41; HYP: 151; TUM: 120; and WHOLE: 132). The LASSO technique was also applied on the combined sets of features extracted from CE and NEC (CE + NEC); from CE, NEC, and HYP (CE + NEC + HYP); and from TUM and HYP (TUM + HYP), selecting 130, 135, and 157 features, respectively. The detailed list of the selected features can be found in the Supplementary Table S2.

### 2.2. Classification Performances of the Two ML Algorithms

Good classification results in the training set were obtained by the two ML algorithms, with SVM cross-validated performances being slightly higher than those of RF (Table 2 and Supplementary Table S3). The best one was achieved when the SVM algorithm was built on the features extracted from the combination of the three ROIs (CE, NEC, and HYP): 83.0% ± 5.7% for accuracy; 83.5% ± 8.9% for sensitivity; 82.5% ± 11.8% for specificity; 0.894 ± 0.056 for AUC (Supplementary Table S3).

However, when the SVM and RF algorithms were tested on the test cohort of 81 patients, their performances dropped substantially (Table 3 and Supplementary Table S4). Both algorithms achieved significant results only using features extracted from the whole lesion (WHOLE), obtaining 64.2% (95% CI: 52.8–74.6%) and 61.7% (95% CI: 50.3–72.3%) accuracies, respectively (Table 3); using the other ROIs, the accuracies were lower than 60%. The top three important features for the “WHOLE” ROI were all extracted from FLAIR images after filtering: GLRLM long run high gray-level emphasis, GLSZM gray-level non-uniformity normalized, and GLSZM low gray-level zone emphasis (Supplementary Figure S1). The top 10 important features contributing the most to the classification are reported, for each ROI, in Supplementary Table S5. Of note, among the 10 most important features extracted from the WHOLE subregion, 8 were from the 3D-FLAIR (4 GLSZM, 2 GLRLM, and 2 first-order features) and 2 from the 3D-T1c (the minimum and median values of the ROI computed on the wavelet-transformed T1 image).

Finally, when age and sex were added to the selected radiomic features, the accuracies obtained by the two algorithms did not change significantly in the test set (Supplementary Figure S2).

## 3. Discussion

In our study, we used a large homogeneous series of GB patients to predict MGMTp methylation status using radiomic features extracted from conventional MRI techniques. Additionally, we compared the classification performance of radiomic models based on features extracted from the entire tumor and from singular tumoral subregions, rarely performed in a large subset of patients. Although some of the previous studies focusing on GBs obtained apparently promising results (AUC values of above 0.80), many of them had important limitations, for instance, the use of cross-validation without an independent validation cohort for performance evaluation [29,30] or a small number of patients [31].

We obtained a high accuracy value on the training set (performing cross-validation and with an AUC value of up to 0.89; Supplementary Table S1), similar to previous analyses on comparable groups of patients, with AUC values between 0.84 and 0.90 [29,30,32,33].

A decrease in the accuracy values is expected when the algorithm is applied to a separate test set [34], as our work confirmed. When applying the trained model to the test set, our best classification performance dropped (AUC = 0.59). Similarly, previous studies that performed a validation phase obtained AUC values between 0.62 and 0.67 [25,35,36,37]. In particular, the study with the largest cohort of patients (*n* = 418) reached an AUC value of 0.65 on the test set [25] and values of sensitivity and specificity (47% and 78%, respectively) that were comparable to those of our study. Our findings confirm and extend their results, as we analyzed further tumor subregions—also including necrosis and combinations of subregions—with a 3D ROI analysis, performed only by a few groups before [25,32,38]. Interestingly, we found that considering the whole tumor area (“WHOLE”) yielded higher classification performances than single components, such as contrast-enhanced or hyperintensity areas.

Three studies that performed external validation achieved higher classification performances, with AUC values between 0.81 and 0.92 [20,31,39]. Two of them [31,39] had a small test set (20 and 28 patients, respectively); in contrast, a third study by Li and colleagues [20] achieved an AUC value of 0.88 with a population of 193 GB patients (60 in the test set). The difference with our results could be due to the inclusion of 2D pre-contrast T1-weighted images in their model, rarely performed in the literature; the addition of this sequence could provide further information to the classification algorithm because tumor heterogeneity is differently represented on pre-contrast and post-contrast T1-weighted images.

Moreover, several factors could be responsible for these heterogeneous results. Radiomic analyses are characterized by multiple steps that can be modified during the pre-processing or the modelling phase [40]. Therefore, this great variability leads to an enormous combination of possible analysis pipelines, making every study substantially unique in the methodological choices. Our pipeline complies with most of the indications of various working groups with expertise in radiomics [40]. With regard to pre-processing, we followed the indications of the IBSI criteria [41] and RQS guidelines [40].

**Figure 2:**
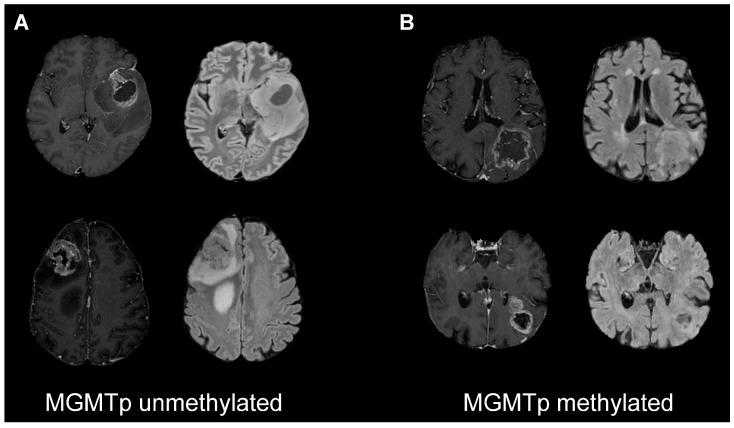
Post-contrast T1-weighted and FLAIR images of four representative patients with MGMTp-unmethylated (**A**) and -methylated (**B**) tumors. The selected patients differed in the combinations of the three most important features for classification: high values of GLRLM long-run high gray-level emphasis ((**A**) MGMTp-unmethylated tumors), high values of GLSZM gray-level non-uniformity normalized ((**A**) MGMTp-unmethylated tumors), and high values of GLSZM low gray-level zone emphasis ((**B**) MGMTp-methylated tumors). Abbreviations: MGMTp = O6-methylguanine-DNA methyltransferase promoter; FLAIR = fluid-attenuated inversion recovery; GLRLM = gray-level run length matrix; GLSZM = gray-level size zone matrix.

Regarding the most important features used for classification, considering our best performance (on the “WHOLE” ROI), the top three were all extracted from FLAIR images. In particular, MGMTp-unmethylated tumors had higher values of the GLRLM long run high gray-level emphasis feature, higher values of the GLSZM gray-level non-uniformity normalized feature, and lower values of the GLSZM low gray-level zone emphasis feature with respect to MGMTp-methylated tumors (Supplementary Figure S1). These three radiomic features describe the grade of homogeneity and the extension of FLAIR hyperintensities (Figure 2). This evidence could suggest that larger lesions with a more homogeneous and higher T2 signal are more likely to be MGMTp-unmethylated. This is in agreement with the results of previous studies based only on the radiological assessment of conventional magnetic resonance images, which showed that MGMTp-unmethylated GBs seem to have a larger amount of edema [9,10]. On the other hand, the most important features for the predictive models did not confirm the role of 3D-T1c sequences, as previously described [9,10].

Due to the low classification performance, the clinical implementation of the present model is not feasible. As mentioned before, this is in agreement with the results of most previous studies. In our opinion, to eventually achieve a clinical use, high sensitivity or specificity values have to be reached. A definitive assessment of the clinical utility of this method could be established by collecting and analyzing larger datasets from different institutions and with wider multimodal imaging modalities.

Our study has some limitations. Regarding the segmentation phase, the sources of variability could be represented by the number of operators (two in our case) who performed the segmentation. We did not perform an inter-observer reliability analysis. We considered only two MRI modalities (3D-T1c and 3D-FLAIR), while other studies also used different techniques, including advance imaging [32,42]. We considered only the classical appearance of GBs that presented with contrast enhancement, necrosis, and infiltrating components, excluding tumors that lacked one of these. We did not perform an external validation with images from other institutes, and we used patients’ data acquired only in our center. However, the use of exams acquired with two different scanners may partially temper this limitation. Lastly, the MGMTp methylation status was analyzed only on a part of the tumor and, therefore, these data could not describe the possible intra-tumoral heterogeneity. In conclusion, in accordance with the results of many previous studies, our results provide some insights into the presence of an association between MRI features and MGMTp methylation status. However, in contrast with other published studies, the association is not sufficiently robust to enable the generation of machine learning classification models for reliable prediction. Further verification is needed to solve the controversial and discordant findings achieved in the literature before a possible translation into clinical practice can be considered.

## 4. Materials and Methods

Our study followed the Radiomic Quality Score (RQS) [40] and Image Biomarker Standardization Initiative (IBSI) [41] guidelines, as recommended by previous works [43,44]. Moreover, the reporting of the present study followed the Transparent Reporting of a multivariable prediction model for Individual Prognosis Or Diagnosis (TRIPOD) and the Must Artificial Intelligence Criteria-10 (MAIC-10) checklists [34,45]. A brief description of these four guidelines/checklists is provided in Table 4. The present study was approved by the local ethics committee of the Fondazione IRCCS Istituto Neurologico Carlo Besta. Informed consent for study participation was waived due to the retrospective nature of this study.

### 4.1. Patient Selection

**Figure 3:**
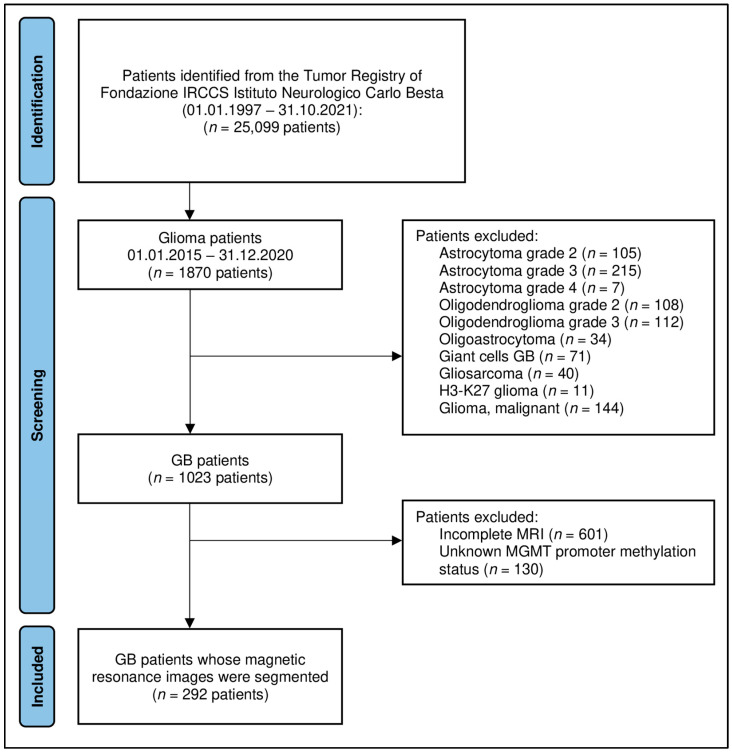
Flowchart of the patient selection. Abbreviations: GB = glioblastoma; MGMT = O6-methylguanine-DNA methyltransferase; MRI = magnetic resonance imaging.

We retrospectively searched for glioma patients recorded in the Tumor Registry of the Carlo Besta Neurological Institute (Milan, Italy) from January 2015 to December 2021. Inclusion criteria were: patients with (i) newly diagnosed, histologically confirmed IDH-wildtype GB (WHO 2021 central nervous system tumor classification); (ii) pretreatment MRI, including 3D-T1 weighted-imaging gadolinium contrast-enhanced (3D-T1c) and 3D-T2/FLAIR (fluid-attenuated inversion recovery, 3D-FLAIR); and (iii) available MGMTp methylation status. For all patients with a known MGMTp methylation status, IDH mutation was also recorded. A total of 1870 patients were screened; 292 patients were finally recruited for the study (Figure 3). Other clinical variables were recorded, including age, sex, Karnofsky performance status (KPS), and overall survival.

### 4.2. MRI Acquisition

All scans were acquired with one of two 1.5 T MRI systems (Scanner 1: Siemens MAGNETOM Avanto, Siemens Healthineers, Erlangen, Germany; Scanner 2: Philips Ingenia, Philips Healthcare, Eindhoven, The Netherlands). Volumetric 3D-T1c and 3D-FLAIR (section thickness 1 mm) were acquired. In all the exams, the contrast agent was power-injected at 5 mL/s followed by a 20 cm^3^ saline chaser at the same flow rate. The contrast agent was gadolinium at 0.1 mmol/kg (gadobutrol, Gadovist, Bayer HealthCare Pharmaceuticals, Berlin, Germany). Scanner 1 parameters were as follows: 3D-T1c (TR: 1160 msec; TE: 4.14–4.24; TI: 600 msec); 3D-FLAIR (TR: 5000–6000 msec; TE: 350–474 msec; TI: 1800–2100 msec). In Scanner 2, different sequences were used across years, with variable sets of parameters: 3D-T1c (TR of 6.5–7.2 msec and TE of 3.2–3.4 msec; TR of 8.1–8.3 msec and TE of 3.7–3.8 msec; TR of 9.3–10.0 msec and TE of 4.6 msec); 3D-FLAIR (TR of 4800 msec, TE of 270–310 msec, and TI of 1650–1660 msec; TR of 8000 msec, TE of 320 msec, and TI of 2400 msec).

### 4.3. MGMT Promoter Methylation Status Testing

Methylation patterns in the CpG islands of *MGMT* were determined using pathological specimens after surgery by the chemical modification of unmethylated cytosines to uracils, followed by methylation-specific polymerase chain reaction (PCR) using primers specific for either methylated or modified, unmethylated DNA [46]. The treatment of tumor DNA (250 ng) with sodium bisulfite was performed with the EpiTect Bisulfite Kit (Cat. No 59104, QIAGEN, Hilden, Germany), following the protocol of the manufacturer. The PCR products were separated by capillary electrophoresis using a 3130 Genetic Analyzer (Applied Biosystems, Foster City, CA, USA) and quantitated using the GeneMapper Software v4.0 (Applied Biosystems, Foster City, CA, USA). The peak height ratio of the PCR products derived from the methylated or unmethylated DNA of the same tumor was calculated. Values > 0.1 were scored as evidence of the methylated status of the *MGMT* promoter [13]. All these procedures were conducted while blinded to information about the radiomic features, which were obtained only in a subsequent phase of the study.

### 4.4. Image Analysis and Modeling Pipeline

Our workflow consisted of the following steps, as recommended by previous works [40,47] and illustrated in Figure 1: (i) the images were collected and pre-processed; (ii) the tumor subregions were manually segmented; (iii) radiomic features were extracted from each sequence and subregion, harmonized and selected; and (iv) two classification algorithms were trained and validated based on the harmonized selected features. Further details for each single step are provided below.

#### 4.4.1. Data Curation: Image Pre-Processing

The Advanced Normalization Tools algorithm was applied in order to correct the bias field distortion and the magnetic field inhomogeneities [48]. For each patient, a 3D-FLAIR image was co-registered with the 3D-T1c, after the removal of the skull through an automatic “deskulling” process. The skull removal phase was performed using the Statistical Parametric Mapping (SPM12, version 7771 https://www.fil.ion.ucl.ac.uk/spm/ [accessed on 20 November 2023]) software, segmenting the brain into the three tissue classes (gray matter, white matter, and cerebrospinal fluid) and obtaining a mask given by the logical sum of the three masks digitally filled in order to remove possible holes. Isotropic voxel resampling was not necessary because the images were already acquired with isotropic voxels (1 × 1 × 1 mm). Image intensity normalization was performed using a z-score transformation, in order to obtain standardized intensity ranges for each imaging modality across all subjects, to generate well-defined inputs for quantitative radiomic feature calculations.

#### 4.4.2. ROI Definition

Tumor subregional segmentation was performed by one neuroradiology resident (M.A.) or by one neuroradiologist (F.M.D.) with 8 years of experience and reviewed by another neuroradiologist (M.M.) with 9 years of experience, blind to the MGMTp methylation status of the patients. Using 3D-T1c images, the following regions were segmented: enhancing tumor volume (CE) and necrotic volume (NEC). Using 3D-FLAIR images, the peritumoral hyperintensity volume (HYP) was segmented. A semi-automatic software was used for segmentation (ITK-SNAP software, version 4.0.1 [49], http://www.itksnap.org/ [accessed on 20 November 2023]), with manual corrections of over- and under-segmentation errors. Neuroradiologists were blinded to clinical or pathological data. An example of tumor segmentation is shown in Figure 1. Two additional ROIs were obtained by merging the segmented subregions: tumor core (TUM) was defined as the union of NEC and CE and whole tumor (WHOLE) was defined as the union of all the three manually segmented areas.

#### 4.4.3. Radiomics: Feature Extraction and Selection from Multiregional and Multiparametric MRI

We used PyRadiomics (version 2.1.2) [50] to extract three groups of radiomic features (14 “shape” features, 18 “first-order statistics” features, and 73 “texture” features) from 3D-T1c and 3D-FLAIR images within the five aforementioned ROIs. Texture features were based on gray-level co-occurrence matrix (GLCM), gray-level run length matrix (GLRLM), gray-level size zone matrix (GLSZM), neighboring gray-tone difference matrix (NGTDM), and gray-level dependence matrix (GLDM). All features were extracted with 3D analysis and 32 fixed bin counts from the native image dataset and, except for shape features, also from wavelet-filtered images (8 derived images) and applying the Laplacian of Gaussian (LoG) filter (5 derived images, with parameter sigma values equal to 1, 2, 3, 4, and 5). In total, 2562 radiomic features were extracted for both MRI modalities and for each of the five ROIs. The steps of the analysis described below were conducted on the set of features extracted for both MRI modalities and each of the five ROIs independently, as well as combining multiple sets of features extracted from CE and NEC (CE + NEC); from CE, NEC, and HYP (CE + NEC + HYP); and from TUM and HYP (TUM + HYP).

The patients’ data were divided into a training set (70%) and a test set (30%), stratified in order to have (i) a similar proportion of patients with MGMTp methylation and (ii) a similar proportion of patients acquired from each MRI scanner. Then, radiomic features in the training set were harmonized using the ComBat technique, which was introduced in the context of the batch normalization of the genetic data acquired from multiple sites and recently adapted for the harmonization of multivendor neuroimaging studies [51,52]. ComBat assumes that the data within each batch conform to a normal distribution and calculates batch-specific location and scale parameters. It subsequently performs an empirical Bayes adjustment to align the data distributions of different batches. The ComBat procedure was conducted separately for 3D-T1c and 3D-FLAIR to obtain sequence-specific estimates of the ComBat transformation.

Kruskal–Wallis test was used to investigate which radiomic features differed among the groups of patients scanned using different MRI sequences, after the ComBat harmonization. After multiple comparison correction using the Benjamini–Hochberg method (i.e., controlling the false discovery rate, FDR), the features that exhibited significant differences (i.e., FDR-adjusted *p*-value of the Kruskal–Wallis test < 0.05) were excluded from the subsequent analyses.

The final feature selection was performed on the training set using a multiple logistic regression model with least absolute shrinkage and selection operator (LASSO) regularization [53]. The key impact of LASSO regularization in feature selection is that it tends to shrink the coefficients of less important features towards zero. As a result, LASSO can be used to automatically select a subset of relevant features while setting others to zero, effectively performing feature elimination. The value of the regularization parameter λ controls the strength of the regularization, with higher values leading to stronger feature shrinkage and selection. The optimal λ value that maximized the balanced accuracy (i.e., the average of sensitivity and specificity) was determined with a 5-fold cross-validation procedure that was repeated 3 times, over a range of possible values (λ = 2^−12^, 2^−11^, …, 2^−1^).

The selected features of the test set were first transformed using the ComBat parameters estimated using the training set. The test dataset was subsequently used to compute the classification accuracy of the models trained on the training set, as described in the following section.

#### 4.4.4. Modelling

We built two machine learning algorithms to classify the presence of MGMTp methylation based on the radiomic features selected for each of the five aforementioned ROIs and their combinations. Specifically, we built a support vector machine (SVM) classifier with a radial kernel [27] and a random forest [28] (RF) using the training set, with a 5-fold cross-validation procedure that was repeated 3 times to determine the optimal model hyperparameters (SVM: “sigma” and “C”; RF: “mtry”) that maximize the balanced accuracy.

We also inspected the importance of each variable in contributing to the prediction of the RF algorithm by computing the average scaled (between 0 and 100) class-specific accuracy decrease. Next, the final SVM and RF classifiers with optimal hyperparameters were tested on the held-out test set to evaluate the classification performance, computing the accuracy, sensitivity, specificity, and area under the receiver operating characteristic (ROC) curve (AUC). Moreover, the two classifiers were built, including patient’s age and sex along with the selected radiomic features, to verify if their contribution improves the classification performance.

All statistical analyses were conducted using the R software, version 4.2.1; in particular, we used package “caret” (version 6.0-92) to perform the feature selection with LASSO and to train the two machine learning algorithms, and the package “NeuroCombat” version 1.0.13 (https://github.com/Jfortin1/neuroCombat_Rpackage [accessed on 20 November 2023) to perform the ComBat harmonization. The statistical significance level was set at *p*-value < 0.05.

## Figures and Tables

**Table 1:** Demographics and clinical characteristics of the patients in the training and test sets. Data are reported as the mean (standard deviation) [range], unless otherwise specified. Abbreviations: M = male; F = female; MGMTp = O6-methylguanine-DNA methyltransferase promoter; KPS = Karnofsky performance status; MRI = magnetic resonance imaging.

Characteristic	Training Set (*n* = 196)	Test Set (*n* = 81)	*p*-Value ^1^
Age, years	61 (11) [22–83]	61 (12) [33–83]	0.942
Sex (M/F)	120/76	51/30	0.892
Overall survival, months	13.2 (10.6) [0.07–51.3]	12.6 (9.9) [0.10–46.1]	0.753
MGMTp (meth./non-meth.)	86/110	35/46	0.999
KPS ^2^, median (range)	90 (50–100)	80 (40–100)	0.225
MRI system (Scanner 1/Scanner 2)	60/136	24/57	0.999

^1^ *p*-values of the comparisons between the training and test set characteristics were computed using Wilcoxon’s rank-sum test for the continuous variables and Fisher’s exact test for the categorical variables. ^2^ KPS was not available in 19 and 8 patients of the training and test sets, respectively.

**Table 2:** Summary of the classification performances on the training set. Data are reported as the mean (SD). Classification metrics were computed on the held-out set for 15 resamples (5-fold cross-validation repeated 3 times) during training. Abbreviations: CE = contrast enhancement; NEC = necrosis; HYP = hyperintensity in FLAIR; TUM = tumor core (union of CE and NEC); WHOLE = whole lesion (union of CE, NEC, and HYP); SVM = support vector machine; RF = random forest; SD = standard deviation; AUC = area under the curve.

Classification Metric	WHOLE		CE		NEC		HYP		TUM	
	SVM	RF	SVM	RF	SVM	RF	SVM	RF	SVM	RF
Accuracy, %	61.4	56.6	63.1	58.5	67.6	58.5	60.9	55.9	61.6	56.6
(SD)	(6.9)	(6.0)	(10.0)	(6.9)	(9.0)	(7.2)	(6.9)	(10.3)	(5.0)	(7.4)
Sensitivity, %	30.2	29.0	54.1	37.3	59.6	43.5	22.4	26.3	36.5	32.9
(SD)	(19.0)	(12.7)	(15.6)	(12.3)	(9.9)	(8.6)	(18.0)	(10.9)	(18.6)	(11.9)
Specificity, %	85.3	77.7	70.1	74.8	73.8	69.8	90.5	78.7	80.7	74.8
(SD)	(13.3)	(7.4)	(16.5)	(9.8)	(14.0)	(11.5)	(8.8)	(14.4)	(15.2)	(10.1)
AUC	0.661	0.535	0.688	0.622	0.759	0.615	0.610	0.549	0.669	0.588
(SD)	(0.120)	(0.100)	(0.104)	(0.090)	(0.069)	(0.087)	(0.124)	(0.100)	(0.112)	(0.081)

**Table 3:** Summary of the classification performances on the test set. Data are reported as the mean (SD). Classification metrics were computed on the 81 patients assigned to the test set. Abbreviations:; CE = contrast enhancement; NEC = necrosis; HYP = hyperintensity in FLAIR; TUM = tumor core (union of CE and NEC); WHOLE = whole lesion (union of CE, NEC, and HYP); SVM = support vector machine; RF = random forest; CI = confidence interval; n/N = numerator/denominator; AUC = area under the curve.

Classification Metric	WHOLE		CE		NEC		HYP		TUM	
	SVM	RF	SVM	RF	SVM	RF	SVM	RF	SVM	RF
Accuracy, %	64.2	61.7	49.4	50.6	50.6	53.1	55.6	53.1	50.6	51.9
(95% CI)	(52.8–74.6)	(50.3–72.3)	(38.1–60.7)	(39.3–61.9)	(39.3–61.9)	(41.7–64.3)	(44.1–66.6)	(41.7–64.3)	(39.3–61.9)	(40.5–63.1)
Sensitivity, %	42.9	37.1	54.3	25.7	48.6	48.6	37.1	25.7	40.0	28.6
(n/N)	(15/35)	(13/35)	(19/35)	(9/35)	(17/35)	(17/35)	(13/35)	(9/35)	(14/35)	(10/35)
Specificity, %	80.4	80.4	45.7	69.6	52.2	56.5	69.6	73.9	58.7	69.6
(n/N)	(37/46)	(37/46)	(21/46)	(32/46)	(24/46)	(26/46)	(32/46)	(34/46)	(27/46)	(32/46)
AUC	0.572	0.594	0.583	0.482	0.453	0.436	0.495	0.543	0.532	0.452
(95% CI)	(0.439–0.705)	(0.464–0.725)	(0.457–0.710)	(0.351–0.613)	(0.323–0.583)	(0.306–0.566)	(0.360–0.630)	(0.414–0.672)	(0.403–0.660)	(0.320–0.583)

**Table 4:** Description of the four guidelines followed by our study. Abbreviations: RQS = Radiomic Quality Score; IBSI = Image Biomarker Standardization Initiative; TRIPOD = Transparent Reporting of a multivariable prediction model for Individual Prognosis Or Diagnosis; MAIC-10 = Must Artificial Intelligence Criteria-10; AI = Artificial Intelligence.

Name	General Description
Radiomic Quality Score (RQS)	It considers radiomics-specific aspects that fall into the following six domains: protocol quality and reproducibility in image and segmentation; feature reduction and validation; biologic/clinical validation and utility; performance index; high level of evidence; and open science [40].
Image Biomarker Standardization Initiative (IBSI)	It provides standardized nomenclature/definitions of image biomarkers, a standardized workflow of image processing, tools for verifying the implementations of radiomics software, and reporting guidelines for radiomics studies [41].
Transparent Reporting of a multivariable prediction model for Individual Prognosis Or Diagnosis (TRIPOD)	It covers a set of recommendations for the reporting of studies developing and/or validating prediction models for both diagnosis and prognosis, for all medical domains, and all types of predictors [34].
Must Artificial Intelligence Criteria-10 (MAIC-10)	It is a short quality assessment tool widely applicable to AI studies in medical imaging focusing on the following aspects: clinical need; study design; safety and privacy; data curation, annotation and partitioning; model description, robustness and explainability; and data transparency [45].

## Data Availability

The datasets generated by the current study are available in the institutional repository of the Fondazione IRCCS Istituto Neurologico Carlo Besta (DOI: 10.5281/zenodo.10410982).

## Supplementary Materials

The following supporting information can be downloaded at: https://www.mdpi.com/article/10.3390/ijms25010138/s1.
