# Supplementary material for: Development of A Radiomic Model for MGMT Promoter Methylation Detection in Glioblastoma Using Conventional MRI
Source: Int J Mol Sci. 2023 Dec 21;25(1):138. doi: 10.3390/ijms25010138 (PMC10778771; doi:10.3390/ijms25010138)
Supplement: Supplementary file 1 [file ijms-25-00138-s001.zip › Supplementary Figure S1.pdf]

Supplementary Figure S1. Comparison of the top 3 selected features for the predictive model based on whole tumor segmentation between MGMT promoter methylated and unmethylated tumors.

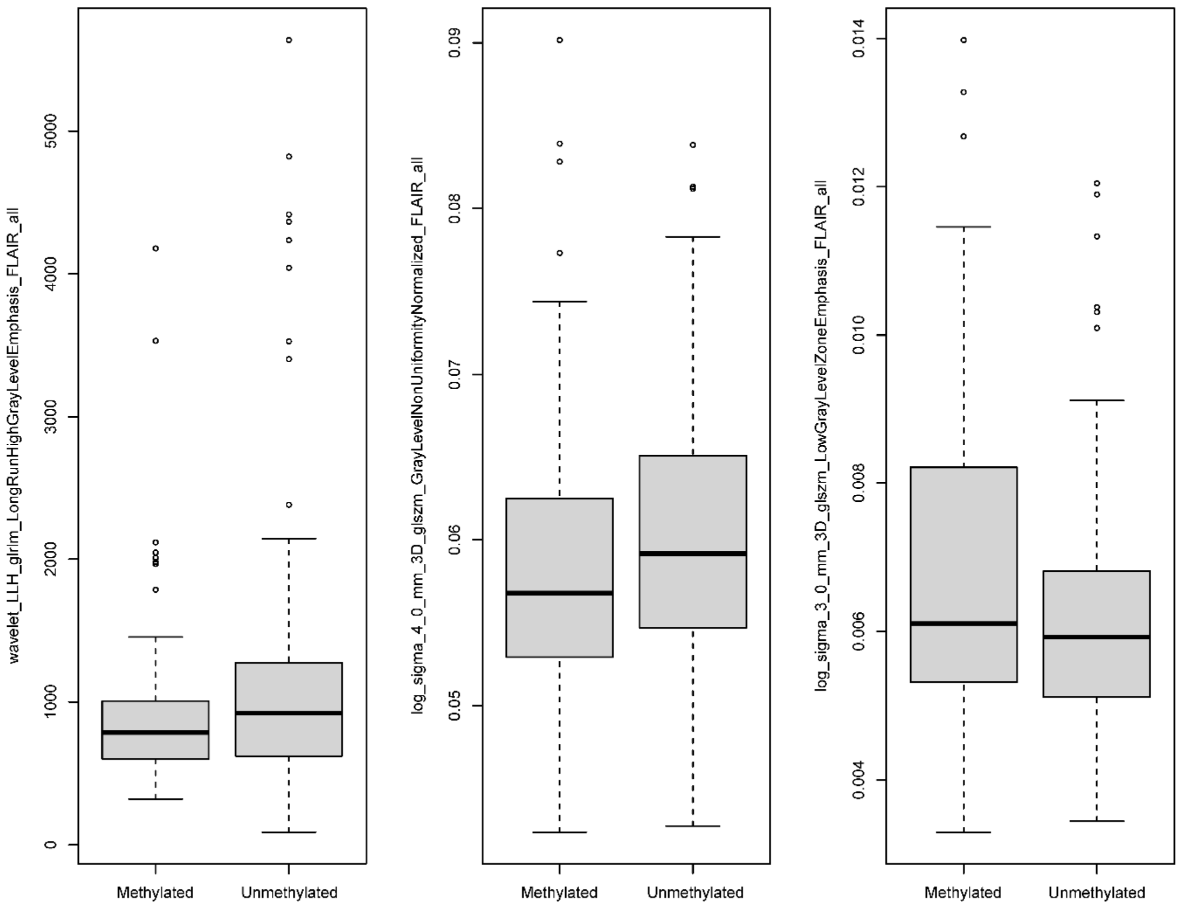

Abbreviations: MGMT = O6-methylguanine-DNA methyltransferase; LoG = Laplacian of Gaussiam filter; L = low pass filter; H = high pass filter; FLAIR = fluid attenuated inversion recovery; GLRLM = Gray Level Run Length Matrix; GLSZM = Gray Level Size Zone Matrix.
