# Supplementary material for: Development of A Radiomic Model for MGMT Promoter Methylation Detection in Glioblastoma Using Conventional MRI
Source: Int J Mol Sci. 2023 Dec 21;25(1):138. doi: 10.3390/ijms25010138 (PMC10778771; doi:10.3390/ijms25010138)
Supplement: Supplementary file 1 [file ijms-25-00138-s001.zip › Supplementary Figure S2.pdf]

Supplementary Figure S2. Comparison of accuracies obtained in the test set by models using radiomic features with or without inclusion of clinical data.

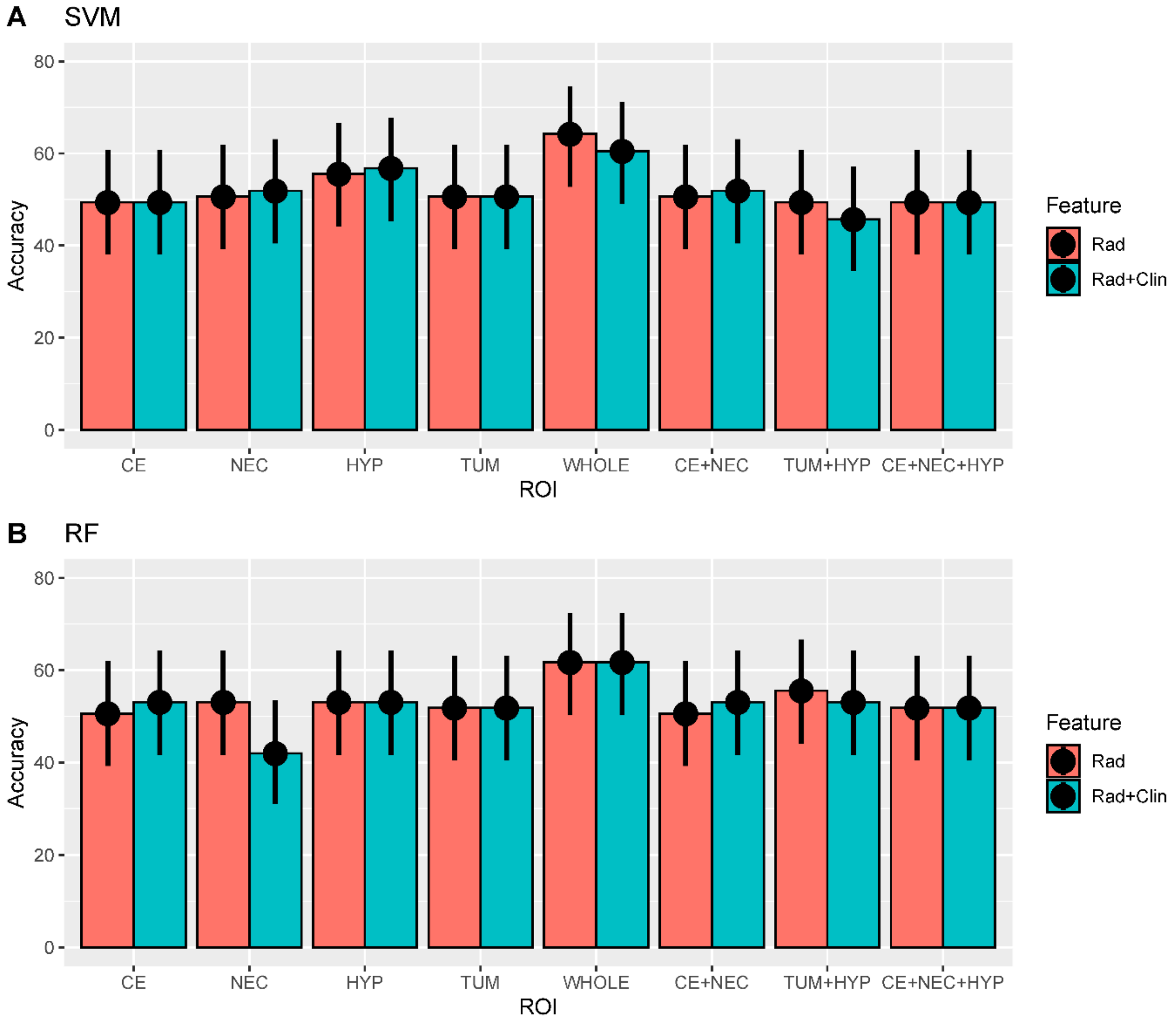

Segments represent 95% confidence intervals. (A) Support Vector Machine. (B) Random Forest.

Abbreviations: SVM = support vector machine; RF = random forest; CE = contrast enhancement; NEC = necrosis; HYP = T2 hyperintensity; TUM = solid tumor; WHOLE = tumor including edema; ROI = region of interest; Rad = radiomic data only; Rad+Clin = radiomic with clinical data.
