# Supplementary material for: Development of A Radiomic Model for MGMT Promoter Methylation Detection in Glioblastoma Using Conventional MRI
Source: Int J Mol Sci. 2023 Dec 21;25(1):138. doi: 10.3390/ijms25010138 (PMC10778771; doi:10.3390/ijms25010138)
Supplement: Supplementary file 1 [file ijms-25-00138-s001.zip › Supplementary Table S3.pdf]

Supplementary Table S3. Summary of classification performances on the training set based on radiomic features extracted from multiple ROIs.

Metric	CE+NEC		TUM+HYP		CE+NEC+HYP	
	SVM	RF	SVM	RF	SVM	RF
Accuracy, % (SD)	71.6 (8.5)	56.8 (7.2)	70.4 (9.4)	61.0 (8.4)	83.0 (5.7)	59.4 (9.8)
Sensitivity, % (SD)	60.4 (17.7)	21.2 (10.6)	64.3 (10.1)	34.1 (10.7)	83.5 (8.9)	31.4 (11.9)
Specificity, % (SD)	80.2 (9.4)	84.0 (7.7)	75.0 (12.2)	81.6 (9.6)	82.5 (8.3)	80.8 (11.8)
AUC (SD)	0.776 (0.085)	0.565 (0.082)	0.785 (0.090)	0.654 (0.122)	0.894 (0.056)	0.637 (0.126)

Data are reported as mean (SD). Classification metrics were computed on the held-out set for 15 resamples (5-fold cross-validation repeated 3 times) during training. Abbreviations: ROI = region of interest; CE = contrast enhancement; NEC = necrosis; HYP = hyperintensity in FLAIR; TUM = tumor core (union of CE and NEC); SVM = Support Vector Machine; RF = Random Forest; SD = standard deviation; AUC = area under the curve.
