# Supplementary material for: Development of A Radiomic Model for MGMT Promoter Methylation Detection in Glioblastoma Using Conventional MRI
Source: Int J Mol Sci. 2023 Dec 21;25(1):138. doi: 10.3390/ijms25010138 (PMC10778771; doi:10.3390/ijms25010138)
Supplement: Supplementary file 1 [file ijms-25-00138-s001.zip › Supplementary Table S4.pdf]

Supplementary Table S4. Summary of classification performances on the test set based on radiomic features extracted from multiple ROIs.

Metric	CE+NEC		TUM+HYP		CE+NEC+HYP	
	**SVM**	**RF**	**SVM**	**RF**	**SVM**	**RF**
Accuracy, % (95% CI)	50.6 (39.3-61.9)	50.6 (39.3-61.9)	49.4 (38.1-60.7)	55.6 (44.1-66.6)	49.4 (38.1-60.7)	51.9 (40.5-63.1)
Sensitivity, % (n/N)	48.6 (17/35)	17.1 (6/35)	51.4 (18/35)	31.4 (11/35)	42.9 (15/35)	28.6 (10/35)
Specificity, % (n/N)	52.2 (24/46)	76.1 (35/46)	47.8 (22/46)	73.9 (34/46)	54.3 (25/46)	69.6 (32/46)
AUC (95% CI)	0.555 (0.429-0.682)	0.473 (0.344-0.601)	0.502 (0.371-0.634)	0.547 (0.418-0.675)	0.505 (0.377-0.633)	0.461 (0.331-0.591)

Classification metrics were computed on the 81 patients assigned to the test set. Abbreviations: ROI = region of interest; CE = contrast enhancement; NEC = necrosis; HYP = hyperintensity in FLAIR; TUM = tumor core (union of CE and NEC); SVM = Support Vector Machine; RF = Random Forest; CI = confidence interval; n/N = numerator/denominator; AUC = area under the curve.
